# Malting of Fusarium Head Blight-Infected Rye (*Secale cereale*): Growth of *Fusarium graminearum*, Trichothecene Production, and the Impact on Malt Quality

**DOI:** 10.3390/toxins10090369

**Published:** 2018-09-11

**Authors:** Zhao Jin, James Gillespie, John Barr, Jochum J. Wiersma, Mark E. Sorrells, Steve Zwinger, Thomas Gross, Jaime Cumming, Gary C. Bergstrom, Robert Brueggeman, Richard D. Horsley, Paul B. Schwarz

**Affiliations:** 1 Department of Plant Sciences, North Dakota State University, P.O. Box 6050, Dept. 7670, Fargo, ND 58108, USA; zhao.jin@ndsu.edu (Z.J.); james.gillespie@ndsu.edu (J.G.); john.barr@ndsu.edu (J.B.); richard.horsley@ndsu.edu (R.D.H.); 2 Department of Agronomy and Plant Genetics, University of Minnesota, Crookston, MN 56716, USA; wiers002@umn.edu; 3 Department of Plant Breeding and Genetics, Cornell University, Ithaca, NY 14853, USA; mes12@cornell.edu; 4 Carrington Research Extension Center, North Dakota State University, P.O. Box 219, Carrington, ND 58421, USA; steve.zwinger@ndsu.edu; 5 Department of Plant Pathology, North Dakota State University, P.O. Box 6050, Dept. 7660, Fargo, ND 58108, USA; thomas.gross@ndsu.edu (T.G.); robert.brueggeman@ndsu.edu (R.B.); 6 School of Integrative Plant Science, Plant Pathology and Plant-Microbe Biology Section, Cornell University, Ithaca, NY 14853, USA; jc2246@cornell.edu (J.C.); gcb3@cornell.edu (G.C.B.)

**Keywords:** rye, variety, environment, type B trichothecenes, Tri5 DNA, malting quality, viscosity, phenolics

## Abstract

This project was initiated with the goal of investigating the malt quality of winter rye cultivars and hybrids grown in the United States in 2014 and 2015, but high levels of deoxynivalenol (DON) were subsequently found in many of the malt samples. DON levels in 75% of the investigated rye samples (n = 117) were actually below 1.0 mg/kg, as quantified by a gas chromatography combined with electron capture detector (GC-ECD). However, 83% of the samples had DON in excess of 1.0 mg/kg following malting, and the average DON level in malted rye was 10.6 mg/kg. In addition, relatively high levels of 3-acetate DON (3-ADON), 15-acetate DON (15-ADON), nivalenol (NIV), and DON-3-glucoside (D3G) were observed in some rye malts. Our results show that rye grain DON is likely a poor predicator of type B trichothecenes in malt in practice, because high levels of malt DON, 15-ADONm and D3G were produced, even when the rye samples with DON levels below 0.50 mg/kg were processed. *Fusarium* Tri5 DNA content in rye was highly associated with malt DON levels (*r* = 0.83) in a small subset of samples (n = 55). The impact of *Fusarium* infection on malt quality was demonstrated by the significant correlations between malt DON levels and wort viscosity, *β*-glucan content, wort color, wort *p*-coumaric acid content, and total phenolic content. Additional correlations of rye *Fusarium* Tri5 DNA contents with malt diastatic power (DP), wort free amino nitrogen (FAN) content, and arabinoxylan content were observed.

## 1. Introduction 

Trichothecene mycotoxins, such as deoxynivalenol (DON), on brewing grains pose a food safety concern to maltsters and brewers, and several studies have shown that between 70 and >100% of the DON present on barley malt can be recovered in the finished beer [1,2,3]. Recent surveys of commercial beers have shown DON and its conjugate, deoxynivalenol-3-glucoside (D3G), to have been present in a large portion of samples representing numerous countries and several continents [3,4,5,6,7,8,9]. Average levels of DON and D3G reported in beer were almost always below 20 μg/L, and, as such, were not considered a safety risk. However, several samples did show levels as high as 500 μg/L DON, which is cause for concern. The results of the above surveys illustrate several important factors. These include the prevalence of the fungal disease, Fusarium head blight (FHB), in many of the important malting barley production regions in North America, South America, and Europe [10], and also illustrates the power of current analytical technology. In addition, the results illustrate the importance of ongoing screening of grain for DON by the malting and brewing industries. As an example, a beer testing at 20 μg/L DON would translate to ≤0.10 mg/kg on the original malt, which is below the limit of quantification (LOQ) of many tests utilized in the grain trade. This hypothetical example assumes 100% transfer of DON from malt to beer and 20 kg/hL malt usage, which is somewhat generous as malt use by the major brewers in the US is closer to 7 kg/hL, while craft beer has been estimated at 27 kg/hL [11]. In addition, a beer formulation is unlikely to utilize only a single malt, and many beers utilize unmalted adjunct in addition to malt.

The behavior of several *Fusarium* species during malting of barley, and the fate of several trichothecenes during the malting and brewing processes, have been studied by a number of researchers, and the topic was recently reviewed [12]. Contamination occurs in the field as a result of FHB, which is most commonly caused by *F. graminearm*, and to a lesser degree by *F. culmorum*, *F. avenaceum*, *F. poae*, and other species. The malting process consists of steeping (soaking), germination, and kilning (drying) phases, and in terms of DON, the common pattern is to see levels decline in steeping. When there is little to no viable *Fusarium* on the grain, DON levels on the finished malt are often lower than the original barley, or even non-detectable. Maltsters generally screen incoming grain for DON, and will reject any lots with DON above a set limit. These limits can change with regions, crop years, and individual companies, but rarely exceed 0.5–1 mg/kg DON. It is also common practice to store infected grain for several months to one year prior to malting, as the viability of *Fusarium* generally declines with storage [13]. However, if viable *Fusarium* is present on the grain when malted, growth and additional DON production can occur during the germination and early kiln phase. A portion of this DON may be converted to D3G through the action of host UDP-glucosyltransferases, which is a host defense response [14]. D3G is not detected by the methodology commonly used in the grain trade, and as such, it has often been referred to as a masked mycotoxin. Finally, high heat during the kilning process kills the *Fusarium* and halts DON production, but DON is thermostable. In cases with viable *Fusarium*, DON levels on the finished malt can be several-fold higher than those observed on the original barley. Increases of up to seven-fold have been reported during laboratory malting [2,12].

Conversions with commercial maltsters [15] have indicated that most barley samples show a relatively rapid decline in the potential for *Fusarium* growth and DON production following harvest and subsequent storage, and the production of DON is not a significant issue. However, there are occasionally samples with aberrant behavior where the potential for DON production in malting does not decline, even after considerable storage time. This seems to be impacted by crop year and growing location, and maltsters in North America sometimes empirically refer to these two cases as external vs. internal infection, respectively. The cause is not known, and the behavior is difficult to predict. Causes could relate to differences in the host and/or pathogen, the timing of infection, and the distribution of the organism within host tissues [12].

Aside from the presence of mycotoxins, the infected kernels can also have pronounced effects on the final malting and brewing quality. This is primarily through the action of pathogen enzymes on the grain [16]. As previously reported [17,18], the activities of *β*-glucanase and xylanase were observed to be higher in the heavily infected barley, and some species of *Fusarium* are capable of producing significant amounts of proteinases. This results in an increase of soluble nitrogen and free amino nitrogen content, darker color, and a lower viscosity of the wort. Nielsen et al. [19] recently reported that the DNA levels of *F. poae* and *F. langsethiae* on barley were correlated with increased wort free amino nitrogen and with decreased extract. Another well-documented effect of *Fusarium* infection is the gushing (spontaneous over-foaming) of bottled beer, which has been associated with the presence of fungal hydrophobins [20].

While the majority of work conducted on *Fusarium* in malting and brewing has been on barley, it must be remembered that the use of other grains, such as wheat and rye, is also important. Wheat malt, in fact, accounts for up to 10% of the total malt use in the USA [11]. A limited number of studies have the evaluated behavior of *Fusarium* species and associated mycotoxins during the malting and brewing of wheat. Krstanović et al. [21] and Spanic et al. [22] inoculated wheat with *F. culmorum,* and followed DON levels through the malting and brewing processes. In the first study, increases in DON levels of 1.8- to 6.4-fold were observed from wheat grain to finished malt, and DON was detected in all beers prepared from contaminated malt samples (n = 5). The second study reported increases in zearalenone (ZEN) in addition to DON. A recent investigation of hard red spring wheat grown in North Dakota (n = 15), reported DON and *Fusarium* Tri5 DNA levels to increase by an average of 4.6-fold and 7.6-fold following malting, respectively [23]. The original DON levels ranged from 0.20–10.13 µg/g.

The use of rye malt in brewing is considerably lower than that of wheat and barley, but it is key in some traditional fermented beverages such as kvass, sahti, and roggenbier, and its utilization in the growing craft beer industry is becoming more common [24]. The use of rye grain and malt in distilling is also experiencing a somewhat of a renaissance. There are limited reports on the malting and/or malt quality of rye, and to the best of our knowledge, no reports on the development of trichothecenes during the malting of rye. A past belief was that rye was less susceptible to FHB than wheat (or barley) [25]. This may relate to the majority of the world’s rye production occurring in northern Europe, where the acreage of the FHB alternative host, maize, has been limited. Schlegel [25], in fact, reported that FHB incidence in rye has increased over the past 10 years, as areas rotated to wheat and maize have increased.

A 1998 survey of Finnish cereal samples (n = 68), showed that rye (n = 43) had contaminated with *Fusarium* spp. (20% infected seeds), and DON (5–111 µg/kg) [26]. Another survey in Thuringia, Germany, showed that 34% of conventionally grown rye samples and 11% of organically grown rye samples contained DON with a mean concentration of 490 µg/kg (total n = 69) [27]. A mixture of *F. graminearum*, *F. culmorum* and *F. avenaceum* isolates were inoculated on 53 commercially grown cultivars of winter and spring triticale, wheat, and rye in Poland, and none of the genotypes were found to be completely resistant to the *Fusarium* infection [28]. The only North American report found that DON was detected on five of the 13 investigated rye samples harvested in the 2010 crop year, and the author stated that FHB on rye has rarely been reported in recent years, probably due to diminishing cultivated acres [29]. 

The objective of the current study was to follow the development of type B trichothecenes, during the malting of rye, to relate these changes to the levels and growth of *F. graminearum*, and to attempt to discern some of the possible impacts of *Fusarium* infection on rye malt quality. The project was actually initiated as a study of rye malt quality, and the high levels of mycotoxins in the malt were only discovered later. While the project was not specifically designed to study *Fusarium* infection and malting, it did provide a unique opportunity for such a large set of samples (n = 117) from two crops years (2014 and 2015) and three rye variety trial locations of Minnesota (MN), North Dakota (ND) and New York (NY) in USA. Samples were naturally infected with *F. graminearum* and several other species, and variable levels of trichothecenes were detected in samples from all locations and years.

While the traditional use of rye is limited when compared to barley, we feel that this topic is important, as the use of rye and rye malt is growing with the increase of the craft malting, brewing, and distilling segments. Craft maltsters that are members of the Craft Malt Guild must use at least 50% of locally produced grains [30], which could present issues in years when FHB is widespread within a region. Craft maltsters generally have a limited capacity for blending samples and sourcing grain from outside their region, when compared to their larger counterparts. In modern practice, malting barley with DON levels of above 0.5 mg/kg have been generally discounted or rejected by malt companies in the USA, but there is no guideline for DON levels in rye to be used for malting and brewing.

## 2. Results and Discussion

### 2.1. Rye Samples and Fusarium Species

The original intent of this work was the evaluation of the malting quality of rye cultivars commonly grown in the USA. However, the high levels of DON, which was later found on many malt samples, rendered the original intent void. This is because of known and potential effects of *Fusarium* infection on quality results [12], and then the associated difficulties with data interpretation. Nevertheless, the samples provided a unique opportunity to assess the behavior of *Fusarium* and trichothecenes during the malting of naturally infected rye, which to our knowledge, has not been previously reported.

The study suffers deficiencies in that it was not specifically designed to evaluate the impact of *Fusarium* infection. As an example, the high DON content of malt samples was only discovered after samples from the 2015 crop had been malted, which was almost 18 months after the 2014 harvest. Had this been known earlier, more samples would have been retained for mycological analysis, and samples would have been plated earlier. On the contrary, the study is robust in the sense that it included naturally infected samples from two crop years (2014 and 2015), three distinct growing regions (MN, ND and NY), and a total of seven test sites (117 samples) (Table S1). The samples were comprised of 29 cultivars/lines which represented most of the types grown in North America. These included open pollinated varieties that are grown for forage, cover crop, or grain, as well as the more recent hybrid grain types. A single cultivar of Triticale was also included in the University of Minnesota trials (n = 4, 2015 crop year).

*Fusarium* species present on a subset (n = 24) of samples from the 2015 crop were determined by morphological identification, and then by translation elongation factor 1-*α* (TEF1a) sequencing, following plating on potato dextrose agar (PDA). One hundred kernels of each sample were plated in four replications. No *Fusarium* was detected on kernels from the five ND samples tested. This may reflect lower levels of infection in ND, but perhaps also the fact that plating was performed more than one year after harvest. In terms of the NY (n = 5) samples, an average of 20% of the kernels yielded *Fusarium* (range: 13–32%). The average for MN (n = 14) samples was 9% (range: 2–18%). In terms of species, the average distribution of colonies from the NY samples was 65% *F. graminearum*, 15% *F. sporotrichiodes*, 5% *F. equiseti* and 12% *F. avenaceum*, respectively. The respective average for the MN samples was 72%, 15%, 11% and 1%, respectively. All samples tested yielded multiple species. *F. graminearum* was predominant on all samples, with the exception of one from MN that showed 50% *F. equiseti* and 25% *F. graminearum.* The *Fusarium* species most frequently isolated from NY rye seed in the current study were also commonly isolated from winter and spring malting barley seed produced in New York State in 2014 through 2017 (Cummings and Bergstrom, unpublished data). Most commonly isolated from barley seed were *F. graminearum*, *F. sporotrichoides*, *F. poae* and *F. equisiti*. A previous report on ND and MN barley showed *F. graminearum* to be predominant, and also the occurrence of the other species reported in the current study [31].

### 2.2. Development of Trichothecenes during the Malting of Rye

**Figure 1:**
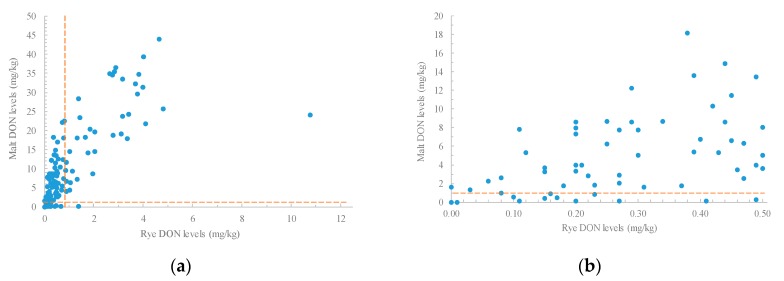
Scattergrams of deoxynivalenol (DON) levels in rye and rye malt samples. (**a**) All samples shown (n = 117, *r* = 0.74, significant level at *p* ≤ 0.05); (**b**) rye samples with DON ≤ 0.50 mg/kg and the corresponding malt samples (n = 64, *r* = 0.52, significant at *p* ≤ 0.05). The dashed line indicates a DON level of 1.00 mg/kg.

When tested, it was found that 55% of the 117 rye samples had DON levels of below 0.50 mg/kg (LOQ), and 73% were below 1.0 mg/kg. Empirical evidence on barley suggests that levels of DON on finished malt were generally lower than the original (unmalted grain) levels when low DON barley was selected [12]. However, the malt results observed in this set of rye samples were dramatic. DON levels in 83% of the malted rye samples were in excess of 1.0 mg/kg (Figure 1a), and increased, on average, by 9-fold following malting. The mean DON level on the malted rye was 10.6 mg/kg, and values as high as 43.9 mg/kg were observed (Table 1). Rye samples from NY and MN had average DON levels of 2.04 and 0.94 mg/kg, respectively. However, DON levels increased to an average of 19.64 mg/kg (NY) and 9.77 mg/kg (MN) in malt, respectively. While levels in the ND samples (2015) were generally below the LOQ, DON was found in 57% of malt samples (0.50–4.00 mg/kg). Similar results also were seen in all the NY (n = 4) and 83% of the MN (n = 43) malted rye samples that had original grain levels below the LOQ (Figure 1b). Only 13% of the total malt samples had DON levels below the LOQ.

In the present study, it appears that a large portion of DON was transformed to D3G during malting (Table 2). The average level of D3G on the rye samples (n = 117) was 0.65 mg/kg, with the highest value being 3.22 mg/kg. However, D3G levels increased by 10-fold on average after malting, and results ranged from the level of detection (LOD) to 20.02 mg/kg. Surprisingly, D3G was detected in all ND malt samples, with mean value of 1.19 mg/kg, though it was generally below 0.10 mg/kg in the unmalted rye. D3G levels increased on average by six- and 14-fold following the malting of NY and MN samples, respectively. However, the ratio of D3G/DON increased from 42 to 60 mol % on average following malting and ranged between 26 and 160 mol % in the malts. The D3G/DON ratio in malts decreased with higher DON on the rye (Table 5). This suggests that there might be a maximum capacity for DON transformation in the germinating grains. This would not be surprising, as excess DON could saturate the available UDP-glucosyltransferases. Our previous report on wheat showed that the average ratio of D3G/DON increased from 23 to 65 mol % following the malting, and the ratios ranged between 34 and 112 mol % in the malted wheat [23]. D3G has been considered to be least toxic, because the conjugation of glucose hinders the capacity of DON interference in protein biosynthesis [32]. However, the toxicity of D3G is still under debate. It has been reported that D3G can be converted back to DON by some human colonic microbiota [33], but also that most of the D3G fed to pigs was recovered in urine and feces [34].

The levels of 3-ADON, 15-ADON and NIV were all below 0.20 mg/kg in all rye samples, but it increased noticeably during malting (Table 3). The average levels of 3-ADON, 15-ADON, and NIV were higher in NY rye malt, at 1.43, 1.34 and <0.50 mg/kg, respectively. The highest level of 3-ADON was 3.97 mg/kg in a NY rye malt, but the highest levels of 15-ADON (5.20 mg/kg) were in a MN rye malt, and NIV (1.81 mg/kg) in another MN rye malt. Such apparent increases of these trichothecenes were not observed in previous reports on malted barley or wheat [12,23]. However, Spanic et al. [22] showed an increase in 3-ADON level in some samples that were inoculated with *F. culmorum.* These results are of interest, as a toxicological study has shown NIV and 15-ADON to be more toxic than DON [35,36]. The 3-ADON was less toxic.

Given the fact that significant amounts of *F. sporotrichiodes* were isolated, the samples should have also been screened for Neosolanol, T-2 and HT-2. Preliminary rescreening of some extracts by LC-MS has suggested the presence of small amounts of HT-2 toxin, but the results remain unconfirmed (Jin and Schwarz, unpublished data). In addition, there is published evidence to suggest poor growth of *F. sporotrichioides* under malting conditions. Habler et al. [37] observed that *F. sporotrichioides* DNA levels decreased during the steeping and did not increase appreciably during germination. Declines in T-2 toxin and HT-2 toxin were observed from barley to finished malt.

The results shown in Table 1, Table 2 and Table 3 were averaged across years, but levels of trichothecenes were generally higher in the 2015 crops in NY and MN than in 2014. ND samples were only from 2015. Further dissection of the environmental response (location and year) can be seen in the principle component analysis (PCA) plots in Figure S1. The score plot (a) showed clear differentiation of the four environments (NY15, NY14, MN15 and MN14). In the loading plot (b), rye and malt trichothecenes were clustered in the third quadrant of PC1 and PC2 that, in total, explained about 61% of the variation. 

### 2.3. Relationships between Levels of Trichothecenes in Rye and Malt

The Pearson correlations between rye and malt trichothecene levels were all significantly different from zero (*p* ≤ 0.001) (Table 4). A moderately strong association was observed between rye and malt DON levels (*r* = 0.74) (Figure 1a) when all samples were evaluated. The correlation coefficients between rye DON and malt D3G, 3-ADON, 15-ADON and NIV levels ranged from 0.52 to 0.67. The coefficients between malt trichothecene levels were all above 0.58, except with malt NIV (*r* = 0.35–0.52). This might be due to NIV levels being below the LOQ in 79% of malt samples. The strength of relationships between rye and malt trichothecene levels suggests that rye with high DON levels may produce malt with high levels of type B trichothecenes. 

To gain a better understanding of the relationships in practical situations, correlations between rye DON and malt trichothecene levels were calculated for rye that fell into the acceptable class that contained original DON ≤ 0.50 mg/kg (n = 64). In this case, the correlation between rye and malt DON was moderate (*r* = 0.52), but the malt DON levels varied from nondetectable to 14.88 mg/kg, with 74% above 1.00 mg/kg (Figure 1b). 

Additionally, the correlation between rye DON and malt 3-ADON, 15-ADON, NIV and D3G ranged from moderately weak to moderate (*r* = 0.36, 0.51, 0.47 and 0.54, respectively). The average levels of 3-ADON, 15-ADON, NIV and D3G were 0.24, 0.58, 0.25 and 4.50 mg/kg, respectively. The maximum levels of 3-ADON, 15-ADON, NIV and D3G were 1.58, 2.49, 0.97 and 14.18 mg/kg, respectively. Only 40% of samples containing 3-ADON, 15-ADON and NIV levels were below the LOD. The results suggest that rye DON is likely a poor predicator of malt trichothecenes in practice, because dramatically high levels of malt DON, 15-ADON, 3-ADON, NIV and D3G were produced even when the rye samples had DON levels of below 0.50 mg/kg. These results highlighted the dramatic increase of type B trichothecenes in the malting of rye. Schwarz et al. [18] micro-malted 125 commercial barley samples harvested from ND in five crop years. The average level of DON was 12.1 mg/kg, and their values ranged from <0.50 to 29.0 mg/kg. However, the average level of DON in malted barley declined to 0.90 mg/kg, with the highest value being 12.0 mg/kg. A significant correlation (*r* = 0.70) between barley and malt DON levels was observed in these samples, but it dropped considerably for barley with DON levels ≤ 1.00 mg/kg (*r* = 0.28). 

### 2.4. Impact of FHB on Grain and Malt Quality of Rye

Infection with FHB is known to impact both grain and malt quality [16]. In the current study, DON levels were used as a marker of FHB infection. A significant relationship was seen between kernel plumpness and rye DON levels (Table 4). However, these results are misleading, and FHB infection is often thought to reduce kernel size [18]. Most of the NY rye samples were hybrid types (Table S1) that have larger kernel size, but NY also had the highest DON levels. ND samples were all open pollinated forage and grain types, with lower kernel plumpness, and there was seemingly less disease pressure (DON levels). MN samples were an intermediate case. As such, the relationships between rye/malt DON levels and thousand kernel weight and plump/thin kernels in this study probably did not have any practical significance.

A strong negative correlation was observed between wort viscosity and DON levels in rye grain and malt (Table 4). Wort viscosity is mainly associated with the content of cell wall *β*-glucans and arabinoxylans, as well as with their molecular weight. The correlations of *β*-glucan and arabinoxylan contents to DON levels in rye and malt were also significantly negative. The significant decrease of wort viscosity and *β*-glucan and arabinoxylan contents was observed with the increase of original rye DON levels in MN and NY samples (Table 5). Unfortunately, the molecular weight of arabinoxylans in wort was not measured in the current study, which has previously been found to affect rye wort viscosity significantly [38]. Previous studies demonstrated that *Fusarium* spp. had the capacity to produce *β*-glucanase and xylanase, and the activities of these hydrolases would degrade cell walls in grains as part of the infection process [16,17].

Significant correlations were also observed between DON levels and wort free amino nitrogen (FAN) content and wort color (Table 4). Wort FAN content increased significantly in MN samples, and wort color increased significantly in NY samples with higher rye DON levels (Table S1). The degradation of protein by the proteinases secreted by *Fusarium* could increase the content of wort FAN, which is known to be a substrate for the Maillard reaction, leading to darker wort color. The results of the current study are in line with previous studies on the malting quality of FHB infected barley [18,19].

An interesting relationship observed in this study was the significant correlations between phenolics and DON. Moderate correlations were observed for wort *p*-coumaric acid, vanillic acid and total phenolic contents to rye and malt DON levels (Table 4). The contents of phenolics in wort increased significantly with initial rye DON levels in both the MN and NY samples (Table S1). It is speculated that this could be caused by *Fusarium* infection, as the digestion of cell walls and possible release of phenolics are part of this process. Cleavage of cross-linked arabinoxylans could explain higher phenolic acid contents [39]. Alternatively, plant pathogens could be eliciting the production of phenolic acids as a defense response in the host [40], which was supported by the significant correlations of malt D3G level to the total phenolic content in wort for both MN and NY samples (*r* = 0.80 and 0.67), respectively (Table 4). However, the general trend of rye and malt quality changes with the increase of DON levels in ND samples was in the opposite in the MN and NY samples. This might be related to the limited numbers of ND samples and the small range of DON levels, as well as their lower germination rate (data not shown).

### 2.5. Relationships between Fusarium Tri5 DNA Levels and Trichotechenes and Malt Quality

**Figure 2:**
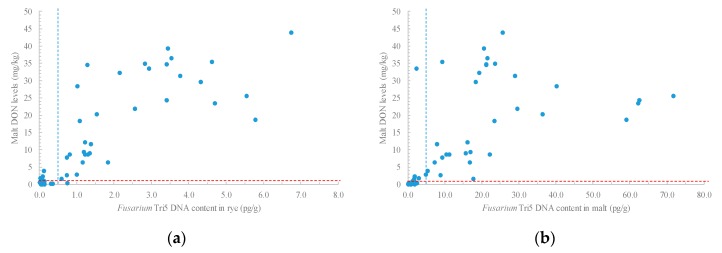
Scattergrams of malt DON and *Fusarium* Tri5 DNA levels in (**a**) rye grain and (**b**) rye malt. The subset (n = 55) of samples was selected to cover the range of malt DON levels. Correlations were significant at *p* ≤ 0.05 for a-rye (*r* = 0.83) and b-malt (*r* = 0.60). The horizontal and vertical dashed lines indicate the levels of 1.00 mg DON/kg and 1.0 pg Tri5 DNA/g, respectively.

*Fusarium* Tri5 DNA was quantified in a subset (n = 55) of rye and corresponding malt samples were selected to cover the range of malt DON levels. DON levels in the original rye samples were all below 5.00 mg/kg and 30 of them were below 0.50 mg/kg. Rye grain and malt Tri5 DNA levels were significantly correlated with trichothecene levels (0.53 ≤ *r* ≤ 0.86) (Table S2). The high correlation of rye Tri5 DNA and malt DON levels (*r* = 0.83) indicated a strong relationship between *Fusarium* infection in original rye to malt DON in this study. Malt DON levels were often in excess of 1.00 mg/kg when the Tri5 DNA content in the original rye samples was above 1.00 pg/g (Figure 2a). The correlation of malt Tri5 DNA and malt DON levels was also significant, but less strong (*r* = 0.60) (Figure 2b). Quantification of DNA levels in the malt could possibly be an underestimate, which could be caused by the partial denaturation of the DNA during kilning, or the loss of *Fusarium* from the malt kernel surface during the physical de-rooting process. 

The effect of *Fusarium* infection on the quality of malted rye was basically in line with what was observed for the correlations between DON and malt quality. Significant relationships between wort viscosity, arabinoxylans, FAN and phenolics also observed with *Fusarium* Tri5 DNA. However, the correlation (*r* = 0.46) between malt Tri5 DNA content and diastatic power (DP) was observed to be significant (Table S2). A significant increase in malt DP was observed, as original rye DON levels increased in both MN and NY samples (Table 5). The reason for this observation is not clear. It could relate to the presence of amylolytic enzymes produced by *Fusarium*, as DP is determined by measuring reducing sugars with a limit-dextrin substrate. Alternatively, it might relate to the release of host *β*-amylase, which is bound to insoluble endosperm protein in sound grain [41].

## 3. Conclusions

The current study demonstrated a very strong potential for the growth of *F. graminearum* and the formation of DON and other type B trichothecenes during the malting of FHB infected rye. The results are rigorous in the sense that they were observed over two crop years, in three distinct growing locations and over a wide range of cultivars. However, it should be stated that the samples were malted just several months following harvest. The viability of *Fusarium* is known to decline with storage of barley [12], but this should be investigated with rye. Further, we do not wish to state that this is the normal case when malting rye and we are unaware of any commercial maltster having encountered this issue. We observed far lower levels of DON in the 2016 rye crops (and malt) from these same regions (Jin and Schwarz, unpublished data) Nevertheless, the potential does exist, and maltsters need to be aware of it when malting rye. Of particular concern is the fact that many rye samples with levels below the LOD developed significant amounts of DON during malting. This is rarely observed with barley.

Although there has been no malting test of rye, wheat and barley from the same locations, we speculate that behavior of *Fusarium* in malting differs between these grains and may relate to the kernel structure and flowering time, as *Fusarium* infection usually takes place during/after flowering stage [42]. The husk threshes free in rye and modern wheats, while it does not in barley. In North America, barley flowering and pollination typically occur just before or during (six to seven weeks) the emergence of the head (inflorescence) from the boot (flag leaf sheath) [43], and infection with *Fusarium* is thought to be most prevalent on the external surface of the grain as the head is largely protected by the sheath. However, in wheat and rye, flowering typically occurs following head emergence. Exposed anthers are especially prone to infection [44]. As such, there would seem to be the possibility for greater ramification within the kernel. A recent report by Góral et al. [45] showed higher content of type B trichothecenes in winter triticale than winter wheat varieties grown in inoculated nurseries (*F. culmorum*) at two locations, despite lower FHB disease symptoms occurring in triticale. Triticale is a hybrid of wheat (*Triticum*) and rye (*Secale*). The observed differences could relate to kernel structure or flowering time.

Finally, the impacts of FHB infection on malt quality observed in this study were quite similar to those previously reported, in that wort viscosity and wort hemicellulose contents were reduced, and wort FAN and color were higher. However, the observation of higher wort phenolic contents has not been previously reported. Again, we speculate that this may be related to the degradation of wort arabinoxylans, or to defense responses of the host.

## 4. Materials and Methods

### 4.1. Rye Samples 

A mix of winter rye samples were obtained from trials conducted at North Dakota State University (North Dakota, ND) (n = 14, two locations), Cornell University (New York, NY) (n = 22), and the University of Minnesota (Minnesota, MN) (n = 81, four locations). NY and MN samples were from 2014 and 2015, whereas ND samples were from 2015 only. Samples represented 24 cultivars and were a mix of forage-cover crop, conventional grain and hybrid grain types (Table S1). The MN sites included one triticale cultivar (n = 4) in 2015. Statistical treatment of the cultivars was not possible as the same cultivars were not grown at all locations, nor always over both crop years. 

### 4.2. Micro-Malting 

Samples were micro-malted approximately two months following harvest in 2014 and 2015. Grain storage was at ambient indoor temperature. Malting conditions were optimized in a previous study [24]. Each sample (80 g, dry basis) was steeped to 45% of moisture and germinated for five days at 16 °C with ≈ 95% relative humidity. Kilning was conducted in stepwise manner from 49 to 85 °C over 24 h. Rootlets were removed from the malt prior to analysis.

### 4.3. Measurement of Malt Quality Parameters

Measurement protocol for all grain, malt, and wort quality parameters were described in a previous report [24]. Rye thousand kernel weight, protein content, kernel plumpness, malt *α*-amylase, DP, extract, wort viscosity, soluble protein, Kolbach index, wort color, FAN and wort *β*-glucan content were analyzed according to the methods of the American Society of Brewing Chemists (ASBC). Wort arabinoxylan content was determined by GC-FID, following hydrolysis with TFA and derivatization of the resultant arabinose and xylose to partially methylated alditol acetates. Phenolic acids in wort were extracted with ethyl ether/ethyl acetate, and then analyzed by quadrupole time of flight liquid chromatography/mass spectrum (Q/TOF LC/MS) (Agilent Technologies, Santa Clara, CA, USA).

### 4.4. Determination of Type B Trichothecenes and D3G

DON, 3-ADON, 15-ADON and NIV were determined according to our standard laboratory procedure [46]. Ground samples (2.50 g) was extracted with 20 mL of acetonitrile/water 84/16, *v*/*v*) for 1 h. Extracts (4.0 mL) were filtered through a C18/alumina (1:3, *w*/*w*) column and an aliquot (2.0 mL) of the supernatant was dried at 55 °C. The dried samples were derivatized with BSA: TMCS: TMSI (3:2:3, *v*:*v*:*v*), and then analyzed on an Agilent 7890N GC-ECD (Agilent Technologies, Santa Clara, CA, USA). Separation was performed on a 35% phenyl siloxane column (30.0 m × 250 µm, 0.25 µm nominal) (Agilent HP-35, Part Number 19091G-133) as previously described. The LOD and LOQ for these trichothecenes were 0.20 mg/kg and 0.50 mg/kg, respectively.

D3G was determined on an Agilent 1290 series liquid chromatograph with a 6540 UHD Accurate-Mass Q-TOF MS as previously described [23]. Extracts (1 mL) prepared for trichothecene analysis prior to the C18/alumina column were filtered through a 0.20 µm nylon filter (Pall Life Sciences, Ann Arbor, MI, USA), and then separated on a ZORBAX Eclipse Plus C18 column (1.8 µm, 2.1 × 50 mm, Agilent, Santa Clara, CA, USA) at 30 °C. Chromatographic conditions and quantification were as previously described The LOD and LOQ were 0.10 mg/kg and 0.50 mg/kg, respectively. 

### 4.5. Identification of Fusarium Species

Sub-samples from the 2015 crops in MN (n = 14), ND (n = 5) and NY (n = 5) were evaluated for the presence of *Fusarium* species. One hundred kernels of each sample were surface-sterilized (1 min 95% ethanol, 1 min 20% sodium hypochlorite, followed by a rinse with sterile water), and then plated onto PDA++ agar (neomycin and kanamycin amended). Plates (25 kernels per plate, with four replications) were incubated at room temp (~24 °C) under black light with a 12 h photoperiod for 5–7 days. All *Fusarium* colonies growing out of each kernel were transferred to individual PDA plates and incubated the same as above. Colonies were identified morphologically and representatives of each that were identified morphologically were confirmed via TEF1α sequencing [47].

### 4.6. Quantitative Analysis of Fusarium Tri5 DNA

The relative amounts of *F. graminearum* present in the rye and malt samples were quantified using a *Fusarium* Tri 5 specific amplicon measured with SYBR Green quantitative real-time polymerase chain reaction (qPCR). The fungal DNA was extracted with a DNeasy Plant Mini Kit (Qiagen Inc. Valencia, CA, USA). The Tri5 specific PCR primer sequences used were TMT_fw (5′-GATTGAGCAGTACAACTTTGG-3′) and TMT_rev (5′-ACCATCCAGTTCTCCATCTG-3′) [19]. The qPCR was performed on a CFX96 Real-time System thermocycler (BIO-RAD, Hercules, CA, USA), with a total reaction volume of 10.0 µL, including 5.0 µL of SsoAdvanced ^TM^ Universal SYBR^®^ Green Supermix (BIO-RAD, Hercules, CA, USA), 3.0 µL of water, 0.50 µL of TMT_fw (10 pmol) and 0.50 µL of TMT-rev (10 pmol) and 1.0 μL of DNA template. The cycling parameters were as follows: initial denaturation for 2 min 45 s at 95 °C, followed by 40 cycles of denaturation at 95 °C for 15 s, and annealing at 62 °C for 30 s. Then melt-curve was generated at the temperature from 65 °C to 95 °C with 0.5 °C increment (5 s per step). The PCR reaction was considered positive if the cycle threshold (CT) value was <40. 

To quantify the initial amount of Tri5 DNA for each sample, the qPCR reactions were performed simultaneously with a serial dilution of purified *F. graminearum* Tri5 PCR amplicon generated with *F. graminearum* DNA template and extracted from single spore cultures grown on V8 media. The serially diluted Tri5 amplicon DNA consisted of seven concentrations; 100 pg, 10 pg, 1 pg, 100 fg, 10 fg, 1 fg and 100 ag.

### 4.7. Data Analysis

The distribution of trichothecenes in rye grain and malt samples was analyzed in ND, NY and MN. Rye and malt quality parameters between different groups, segregated by rye DON level, were compared, and analysis of variance (ANOVA) was used for identifying significant differences (*p* ≤ 0.05). ANOVA was conducted with Origin 8.5 (Electronic Arts Inc., Redwood, CA, USA). Relationships between trichothecenes and rye/malt quality parameters to DON levels were analyzed for all samples (n = 117) using Pearson correlation (SAS 9.4, version 14.0.0, SAS Institute Inc., Cary, NC, USA). Pearson correlations between *Fusarium* tri5 DNA and DON levels were analyzed in a smaller subset (n = 55). The effects of rye variety and environmental factors (crop years and locations) on malt quality were analyzed using principal component analysis (PCA) (Discovering JMP 14^®^, SAS Institute Inc., Cary, NC, USA, 2018).

## Figures and Tables

**Table 1:** Development of deoxynivalenol (DON) during the malting of rye samples grown in New York, North Dakota and Minnesota (2014 and 2015 crop years).

Location	No. of Samples	DON in Rye (mg/kg)						DON in Malt (mg/kg)						
		Mean	Min	Max	Sample (%)			Mean	Min	Max	Sample (%)			
					<0.50	0.50–1.00	>1.00				<0.50	0.50–1.00	1.01–10.00	>10.00
ND ^ab^	14	<0.50 ^c^	<0.20 ^d^	1.37	91	9	0	1.03	<0.20	3.95	43	21	36	0
NY ^a^	22	2.04	<0.50	4.66	23	18	59	19.64	2.04	43.92	0	0	50	50
MN ^a^	81	0.94	<0.20	10.76	54	22	23	9.77	<0.20	28.34	11	1	50	38
Total	117	1.06	<0.20	10.76	55	18	27	10.60	<0.20	43.92	13	3	49	36

^a^ ND, NY and MN were short for North Dakota, New York and Minnesota, respectively; ^b^ Only 2015 for ND samples; ^c^ limit of quantification (LOQ) for DON by the gas chromatography combined with electron capture detector (GC-ECD): 0.50 mg/kg; ^d^ limit of detection (LOD): 0.20 mg/kg.

**Table 2:** Development of DON-3-glucoside during the malting of rye samples.

Location	No. of Samples	D3G in Rye (mg/kg)			D3G in Malt (mg/kg)		
		Mean	Min	Max	Mean	Min	Max
ND ^a^	14	<0.50 ^b^	<0.10 ^c^	<0.50	1.19	<0.50	2.67
NY	22	1.51	<0.50	3.22	11.03	2.81	20.02
MN	81	0.51	<0.10	2.76	7.50	<0.50	14.71
total	117	0.65	<0.10	3.22	7.41	<0.50	20.02

^a^ Only 2015; ^b^ LOQ for D3G detection by LC-MS: 0.50 mg/kg; ^c^ LOD for D3G: 0.10 mg/kg.

**Table 3:** Levels of 3-acetate DON (3-ADON), 15-acetate DON (15-ADON) and nivalenol (NIV) in rye malt samples ^a^.

Location	No. of Samples	3-ADON in Malt (mg/kg)			15-ADON in Malt (mg/kg)			NIV in Malt (mg/kg)		
		Mean	Min	Max	Mean	Min	Max	Mean	Min	Max
ND ^b^	14	<0.20 ^c^	<0.20	<0.50 ^d^	<0.50	<0.20	<0.50	<0.50	<0.20	<0.50
NY	22	1.43	<0.20	3.97	1.34	<0.20	2.78	<0.50	<0.20	0.66
MN	81	0.52	<0.20	2.64	1.18	<0.20	5.20	<0.50	<0.20	1.81
total	117	0.63	<0.20	3.97	1.08	<0.20	5.20	<0.50	<0.20	1.81

^a^ The levels of 3-ADON, 15-ADON and NIV in all rye samples were below the LOD (0.20 mg/kg). ^b^ Only 2015; ^c^ LOD for 3-ADON, 15-ADON and NIV by GC-ECD: 0.20 mg/kg; ^d^ LOQ: 0.50 mg/kg.

**Table 4:** Pearson correlations between trichothecene levels and grain/malt quality parameters ^a^.

Trichothecenes	Rye DON	Malt DON	Malt D3G	Malt 3-ADON	Malt 15-ADON	Malt NIV
Rye DON	1					
Rye D3G	0.81 ***					
Malt DON	0.74 ***	1				
Malt D3G	0.67 ***	0.96 ***	1			
Malt 3-ADON	0.63 ***	0.92 ***	0.85 ***	1		
Malt 15-ADON	0.58 ***	0.80 ***	0.82 ***	0.61 ***	1	
Malt NIV	0.52 ***	0.38 ***	0.35 ***		0.45 ***	1
**Grain quality**						
Rye thousand kernel weight	0.37 ***	0.29 *		0.28 *		0.28 *
Plump kernels	0.36 ***	0.31 **	0.86 ***			
Thin kernels			−0.78 ***			0.33 **
**Malt quality**						
Malt *α*-amylase					−0.32 **	
Malt diastatic power (DP)						
Malt extract				0.24 *		
Wort viscosity	−0.58 ***	−0.64 ***	−0.64 ***	−0.61 ***	−0.61 ***	−0.31 ***
Wort soluble protein						0.31 **
Kolbach index						
Wort color		0.24 *	0.29 *	0.25 *	0.25 *	0.46 ***
Wort free amino nitrogen (FAN)	0.27 *				0.28 *	0.67 **
Wort *β*-glucan	−0.24 *	−0.34 **	−0.38 **	−0.31 **	−0.31 **	
Wort arabinoxylan	−0.27 *					
Wort ferulic acid			0.25 *			
Wort *p*-coumaric acid	0.43 ***	0.70 ***	0.78 ***	0.75 ***	0.75 ***	
Wort vanillic acid	0.31 **	0.27 *		0.28 *		
Wort total phenolic acids	0.41 ***	0.51 ***	0.49 ***	0.46 ***	0.41 ***	

^a^ This analysis is based on samples across all locations and years (n = 117); *, ** and *** indicate significance at *p* ≤ 0.01, *p* ≤ 0.001 and *p* ≤ 0.0001.

**Table 5:** Mean levels of malting quality parameters segregated by location and DON range (ANOVA analysis).

Parameters	ND rye DON (mg/kg)		MN rye DON (mg/kg)			NY rye DON (mg/kg)	
	<0.20	0.20–1.37	<0.50	0.50–2.00	2.01–10.76	<2.00	2.01–4.66
**No. of samples**	**9**	**5**	**44**	**28**	**9**	**11**	**11**
**Trichothecenes**							
Rye D3G (mg/kg)	0.03 a	0.13 a	<0.50 a	0.52 b	1.69 c	<0.50 a	2.57 b
Malt DON (mg/kg)	1.10 a	0.89 a	5.53 a	12.80 b	21.08 c	4.19 a	35.09 b
Malt D3G (mg/kg)	1.39 a	0.82 a	5.34 a	9.45 b	11.98 b	3.78 a	18.29 b
Malt D3G/DON (mol%)	74.88 a	79.47 a	72.85 a	53.51 b	37.15 c	63.64 a	34.01 b
Malt 3-ADON (mg/kg)	<0.20 a	<0.20 a	<0.50 a	0.75 b	0.93 b	<0.50 a	2.56 b
Malt 15-ADON (mg/kg)	<0.20 a	<0.20 a	0.68 a	1.61 b	2.28 c	<0.50 a	2.04 b
Malt NIV (mg/kg)	<0.20 a	<0.20 a	0.26 a	0.42 b	1.04 c	<0.50 a	<0.50 a
**Grain quality**							
Rye thousand kernel weight (g)	30.23 a	29.84 a	27.29 a	30.00 b	33.32 b	30.70 a	30.66 a
Rye Plump percent (%)	49.30 a	59.45 a	46.08 a	62.46 b	77.23 b	73.57 a	66.94 a
Rye Thin percent (%)	2.30 a	6.94 a	14.97 ab	7.80 bc	7.33 c	1.71 a	3.46 b
**Malt quality**							
Malt *α*-amylase (DU)	69.04 a	70.58 a	79.97 a	71.09 b	66.58 b	87.80 a	83.27 a
Malt DP (ASBC)	143.67 a	118.10 b	129.91 a	147.31 ab	180.02 bc	51.45 a	73.25 b
Malt extract (%, db)	83.17 a	83.63 a	83.21 a	83.77 a	82.48 a	88.44 a	88.28 a
Wort viscosity (mPa)	5.54 a	6.23 a	4.57 a	3.66 b	2.17 c	2.83 a	2.36 a
Wort soluble protein (% malt, db)	10.05 a	8.14 b	9.62 a	8.87 a	10.61 a	5.60 a	5.98 a
Kolbach index (%)	67.95 a	66.00 a	66.49 a	63.92 a	68.18 a	67.21 a	70.05 a
Wort color (°SRM)	6.58 a	3.83 b	6.79 a	7.51 a	7.70 a	4.85 a	6.53 b
Wort FAN content (mg/L)	223.42 a	220.11 a	255.10 a	260.79 ab	311.72 c	227.56 a	233.69 a
Wort *β*-glucan content (mg/L)	81.39 a	96.24 a	33.53 a	28.91 ab	18.86 b	15.82 a	3.99 b
Wort arabinoxylan content (mg/L)	234.18 a	215.02 a	251.21 a	251.88 ab	202.13 b	233.57 a	234.32 a
Wort ferulic acid content (mg/L)	8.51 a	6.24 b	9.37 a	9.55 a	8.91 a	11.34 a	10.33 a
Wort *p*-coumaric acid content (mg/L)	0.63 a	0.52 a	1.67 a	2.96 bc	3.09 c	2.69 a	3.66 b
Wort vanillic acid content (mg/L)	1.91 a	1.92 a	3.60 a	2.72 a	5.84 b	3.70 a	6.88 b
Wort total phenolic acid content (mg/L)	11.75 a	9.26 a	15.76 a	16.70 ab	19.58 b	18.73 a	22.28 b

Different letters following the data indicates significant differences (*p* < 0.05) for the parameter within the same location.

## Supplementary Materials

The following are available online at http://www.mdpi.com/2072-6651/10/9/369/s1, Figure S1: Principal component analysis (PCA) of rye cultivar and crop years (2014 and 2015), Table S1: Rye Samples utilized for the evaluation of the impact of FHB infection on malt quality (2014 and 2015 crops years), Table S2: Pearson correlations between malt quality and Fusarium Tri5 DNA content in a subset selected (n = 55) to represent the range in malt DON.
