# Supplementary material for: Malting of Fusarium Head Blight-Infected Rye (Secale cereale): Growth of Fusarium graminearum, Trichothecene Production, and the Impact on Malt Quality
Source: Toxins (Basel). 2018 Sep 11;10(9):369. doi: 10.3390/toxins10090369 (PMC6162642; doi:10.3390/toxins10090369)
Supplement: Supplementary file 1 [file toxins-10-00369-s001.pdf]

Supplementary Materials: Malting of Fusarium Head Blight Infected Rye (Secale cereale): Growth of Fusarium graminearum, Trichothecene Production, and the Impact on Malt Quality

Zhao Jin, James Gillespie, John Barr, Jochum J. Wiersma, Mark E. Sorrells, Steve Zwinger, Thomas Gross, Jaime Cumming, Gary C. Bergstrom, Robert Brueggeman, Richard D. Horsley and Paul B. Schwarz

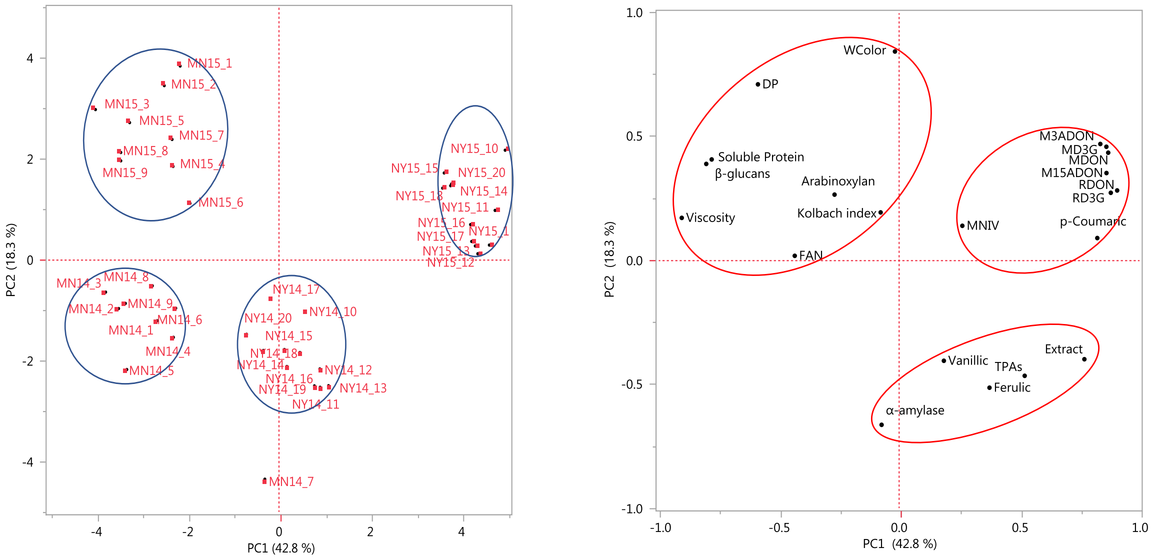

(**a**) (**b**)

Figure S1. Principal component analysis (PCA) of rye cultivar and crop years (2014 and 2015) (**a**) Score plot, (**b**) loading plot. Codes in score plot a are location (MN or NY), crop year (2014 or 2015) and cultivar No. (1–20); there were 11 cultivars grown in NY for both 2014 and 2015, and nine other cultivars grown at Crookston of MN in both crop years. The R Code in loading plot b designates DON and D3G in rye, respectively. The M code designates DON, D3G, 3-ADON, 15-ADON and NIV in malt, respectively.

Table S1. Rye Samples utilized for the evaluation of the impact of FHB infection on malt quality (2014 and 2015 crops years).

Variety and Type Number (n)	Variety by State		
Total Samples (n = 117)	Minnesota [a] (n = 81)	North Dakota [b] (n = 14)	New York [c] (n = 22)
Forage/Cover Crop (n=32) [d]	Aroostook, Elbon, Maton II, Oklon, Wheeler and Wrens-Abruzzi	Aroostook, Hancock and Wheeler	Aroostook
Conventional Grain (n = 42) [d]	Dukato, Musketeer, Prima, Rhymin, Spooner and Tulus [e]	AC Hazlet, Dacold, ND-Dylan, Musketeer, Rhymin, and Spooner	AC Hazlet and Danko
Hybrid Grain (n = 43) [f]	A, B, C, D, E, and F		G, H, I, J, K, L and M

[a] Variety trials in Crookston, Lamberton, Le Center and St Paul, MN. [b] Variety trials in Carrington and Minot, ND. [c] Variety trials in Ithaca, NY. [d] Open pollinated variety. [e] Tulus is a winter triticale. [f] The 13 hybrid cultivars and lines were obtained from commercial seed companies, and are identified by alphabetical designation only.

Table S2. Pearson correlations between malt quality and *Fusarium* Tri5 DNA content in a subset selected (n = 55) to represent the range in malt DON.

	Rye Tri5 DNA	Malt Tri5 DNA
**DNA**		
Rye Tri 5 DNA	1	
Malt Tri 5 DNA	0.74 ***	1
**Trichothecenes**		
Rye DON	0.86 ***	0.64 ***
Malt DON	0.83 ***	0.60 ***
Malt D3G	0.80 ***	0.61 ***
Malt 3-ADON	0.80 ***	0.54 ***
Malt 15-ADON	0.63 ***	0.73 ***
Malt NIV	0.53 ***	0.66 ***
**Malt quality**		
Malt α-amylase		
Malt DP		0.46 **
Malt extract		
Wort viscosity	−0.69 ***	−0.67 ***
Wort soluble protein		
Wort color		
Wort FAN		0.40 *
Wort β-glucan		
Wort arabinoxylan		−0.40 *
Wort ferulic acid		
Wort p-coumaric acid	0.70 ***	0.66 ***
Wort vanillic acid		
Wort caffeic acid	0.67 ***	0.74 ***
Total phenolic acid	0.48 **	

*, ** and *** indicate significant correlations at $p \leq 0.01$, $p \leq 0.001$ and $p \leq 0.0001$.
